# Transplantation of iPS‐derived vascular endothelial cells improves white matter ischemic damage

**DOI:** 10.1111/jnc.14949

**Published:** 2020-01-21

**Authors:** Bin Xu, Masashi Kurachi, Hiroya Shimauchi‐Ohtaki, Yuhei Yoshimoto, Yasuki Ishizaki

**Affiliations:** ^1^ Department of Molecular and Cellular Neurobiology Gunma University Graduate School of Medicine Maebashi Japan; ^2^ Department of Neurosurgery Gunma University Graduate School of Medicine Maebashi Japan

**Keywords:** cell transplantation, endothelial cells, iPS cells, remyelination, white matter infarct

## Abstract

White matter infarct induces demyelination and brain dysfunction. We previously reported that transplantation of brain microvascular endothelial cells improved the behavioral outcome and promoted remyelination by increasing the number of oligodendrocyte precursor cells in the rat model of white matter infarct. In this study, we investigated the effects of transplantation of vascular endothelial cells generated from human induced pluripotent stem cells (iPSCs) on the rat model of white matter infarct. Seven days after induction of ischemic demyelinating lesion by injection of endothelin‐1 into the internal capsule of a rat brain, iPSC‐derived vascular endothelial cells (iVECs) were transplanted into the site of demyelination. The majority of iVECs transplanted into the internal capsule survived for 14 days after transplantation when traced by immunohistochemistry for a human cytoplasmic protein. iVEC transplantation significantly recovered hind limb rotation angle as compared to human iPSC or rat meningeal cell transplantation when evaluated using footprint test. Fourteen days after iVEC transplantation, the infarct area remarkably decreased as compared to that just before the transplantation when evaluated using magnetic resonance imaging or luxol fast blue staining, and remyelination was promoted dramatically in the infarct when assessed using luxol fast blue staining. Transplantation of iVECs increased the number of oligodendrocyte lineage cells and suppressed the inflammatory response and reactive astrocytogenesis. These results suggest that iVEC transplantation may prove useful in treatment for white matter infarct.

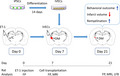

## Graphical abstract

We investigated the effects of transplantation of vascular endothelial cells generated from human induced pluripotent stem cells (iPSCs) on the rat model of white matter infarct. Transplantation of iPSC‐derived vascular endothelial cells (iVECs) ameliorated ischemic demyelinating (DM) lesion in endothelin‐1 (ET‐1)‐treated internal capsule (IC) of a rat brain. iVEC transplantation improved locomotor function when evaluated using footprint (FP) test, and reduced infarct area when monitored using magnetic resonance imaging (MRI). In addition, transplanted iVECs promoted remyelination in the infarct area when examined using luxol fast blue (LFB) staining. Taken together, our results suggest that transplantation of human iPSC‐derived vascular endothelial cells may lead to development of effective therapies for white matter infarct.

**Abbreviations used**
Ac‐LDL: acetylated low‐density lipoprotein
BSA: bovine serum albumin
ET‐1: endothelin‐1
EVs: extracellular vesicles
FBS: fetal bovine serum
GFAP: glial fibrillary acidic protein
HBSS: Hank's balanced salt solution
hESFM: human endothelial serum‐free medium
IC: internal capsule
iPSC: induced pluripotent stem cell
iVEC: iPSC‐derived vascular endothelial cell
LFB: luxol fast blue
LNFP I: lacto‐*N*‐fucopentaose I
LR: limb rotation
MC: meningeal cell
MR: magnetic resonance
MVEC: microvascular endothelial cell
OL: oligodendrocyte
OPC: oligodendrocyte precursor cell
PBS: phosphate‐buffered saline
RRIDs: Research Resource Identifiers (see scicrunch.org)
UM: unconditioned medium
VEGF: vascular endothelial growth factor
vWF: von Willebrand factor

## 1 INTRODUCTION

Ischemic stroke in white matter causes severe neurological disorder and brain dysfunction as a result of interruption of the blood flow (Otero‐Ortega et al., 2015; Wang et al., 2016). Acute‐phase interventions, such as tissue‐type plasminogen activator and mechanical thrombectomy, exist for ischemic stroke with dramatically improved outcomes. After the acute phase of stroke, however, the only available therapy to aid recovery is rehabilitation, but the extent of recovery and outcome may not achieve a level sufficient for functional independence (Love, Selim, Spector, & Lo, 2019). The primary victim of white matter infarct is myelin, which wraps axon and enables rapid communication of neuronal signals among different regions of the central nervous system (Deoni et al., 2011; Marin & Carmichael, 2018). Ischemic stroke leads to extensive demyelination and defective remyelination in white matter (Fumagalli, Lecca, & Abbracchio, 2016; Maki, Liang, Miyamoto, Lo, & Arai, 2013). Therefore, promotion of remyelination in white matter ischemia is critical to the recovery of proper brain functions.

We previously reported that transplantation of brain microvascular endothelial cells (MVECs) improved the behavioral outcome and promoted remyelination in an endothelin‐1 (ET‐1)‐injected white matter infarct model (Puentes et al., 2012). We also found that MVEC transplantation inhibited apoptosis of oligodendrocyte precursor cells (OPCs) (Iijima et al., 2015). Furthermore, we revealed that extracellular vesicles (EVs) secreted from endothelial cells promoted survival, proliferation and motility of cultured OPCs in vitro (Kurachi, Mikuni, & Ishizaki, 2016). Taken together, our previous studies suggest a possibility that vascular endothelial cells may prove useful for treatment of white matter infarct.

There remain, however, two major obstacles to overcome for the use of vascular endothelial cells in clinical application. One is the source of human vascular endothelial cells, and the other is the problem of xenograft rejection. In this regard, human induced pluripotent stem cells (iPSCs) exhibit a great potential in regenerative medicine (Kokubu, Yamaguchi, & Kawabata, 2017). iPS cell stock for regenerative medicine is being established in many countries (Kim et al., 2017). The stock holds iPSCs of guaranteed quality which can be supplied quickly to medical care institutions when required. The iPSCs can be obtained by reprogramming of somatic cells and then enabling the generation of patient‐specific cells for medical treatment (Maherali & Hochedlinger, 2008). Furthermore, transplantation of iPSC‐derived specific cells is expected to evoke no xenograft rejection (Wu, Zhang, Mishra, Tardif, & Hornsby, 2010).

In this study, we examined the effects of transplantation of human iPSC‐derived vascular endothelial cells (iVECs) on white matter infarct. ET‐1 was injected into the internal capsule (IC) to induce infarction, and iVECs were transplanted into the site of demyelination 7 days after induction of infarction. iVEC transplantation significantly recovered motor deficits, diminished infarct area, and promoted remyelination in the ET‐1‐injured IC. These results suggest that iVEC transplantation may prove useful in treatment for white matter infarct.

## 2 MATERIALS AND METHODS

This study was not preregistered.

### 2.1 Animals

Male Sprague–Dawley (*SD*) rats (8 weeks old; 180–220 g weight) (Research Resource Identifiers, RRID: RGD_70508) were obtained from SLC. All experiments were performed in accordance with the guidelines for Animal Experimentation at Gunma University Graduate School of Medicine and were authorized by the Gunma University Ethics Committee (Permit Number: 18‐016). To minimize animal suffering, all experiments were conducted in compliance with the ARRIVE guidelines. Additionally, rats were allowed to recover for 2 hr in a 28°C room after operation, and animals were deeply anesthetized to reduce the suffering before killing. All rats were housed in standard rat cages with a total of two animals per cage and adaptively fed for 3–5 days before the initiation of the study in a temperature‐controlled room with a 12‐hr light‐dark cycle and were fed on a standard diet ad libitum with free access to water. No randomization was performed to allocate rats in this study. Rats were arbitrarily assigned to experimental groups. All experimental procedures were carried out between 10 a.m. and 9 p.m. All efforts were made to use only the number of animals necessary to produce reliable results.

### 2.2 Cell culture

Human iPS cell line (IMR90‐4, WiCell) (RRID: CVCL_C437) was maintained on growth factor‐reduced Matrigel (BD Bioscience, Cat# 356230) in mTeSR1 culture medium (STEMCELL Technologies, Cat# ST‐85850). iPSCs were passaged every 4–6 days with Versene (Gibco, Cat# 15040066) at a typical ratio of 1:10. Although iPSCs have an infinite lifespan, we generally passaged the cells less than 10 times, because the stress associated with passaging methods and culture conditions causes chromosomal instability and differentiation bias. The cell line was authenticated recently by a STR profile analysis on July 5, 2017 (WiCell). Furthermore, it is not on the list of commonly misidentified cell lines by the International Cell Line Authentication Committee (ICLAC; http://iclac.org/databases/cross-contaminations/).

Meningeal cells (MCs) were prepared from 8‐week‐old rat cerebra as previously described (Puentes et al., 2012). Twenty rats were killed by decapitation under deep anesthesia with isoflurane. Meninges were peeled carefully from the cerebral cortices. Then the tissues were digested with 0.125% trypsin (Sigma, Cat# T9935) in Hank's balanced salt solution (HBSS; Fujifilm Wako Pure Chemicals, Cat# 085‐09355) for 10 min at 37°C, followed by mechanical trituration with pipettes and centrifuged. The dissociated cells were re‐suspended in Dulbecco's modified Eagle medium (DMEM; Fujifilm Wako Pure Chemicals, Cat# 043‐30085) with 10% fetal bovine serum (Gibco, Cat# 12483‐020), plated onto tissue culture dishes (Corning), and maintained in 5% CO_2_ at 37°C.

### 2.3 iVEC differentiation

Differentiation of iVECs from iPSCs was performed as previously described (Kokubu et al., 2017; Lippmann, Al‐Ahmad, Azarin, Palecek, & Shusta, 2014; Lippmann et al., 2012; Stebbins et al., 2016). Briefly, iPSCs were dissociated with Accutase (Millipore, Cat# SCR005) for 5 min, and the number of live cells was quantified with a hemacytometer using a trypan blue stain (Fujifilm Wako Pure Chemicals, Cat# 207‐03252). Singularized iPSCs were seeded on a Matrigel‐coated 6 well plate (Corning) at a density of 10,000 cells/cm^2^ in mTeSR1 medium supplemented with 10 μM Y27632 (ROCK inhibitor; Fujifilm Wako Pure Chemicals, Cat# 036‐24023) for the first 24 hr to promote cell attachment. After 24 hr, Y27632 was withdrawn by replacing mTeSR1 medium. The medium was changed daily for 2 days. When the optimal density (30,000 cells/cm^2^) was reached (25,000–40,000 cells/cm^2^ is acceptable), the medium was replaced with unconditioned medium (DMEM/F12 (1:1) (Gibco, Cat# 11330032) containing 20% knock‐out serum replacement (KOSR, Gibco, Cat# 10828010), 1% MEM (minimum essential medium) non‐essential amino acids (Gibco, Cat# 11140050), 0.5% GlutaMAX (Gibco, Cat# 35050‐061), and 0.1 mM β‐mercaptoethanol (NACALAI TESQUE, Cat# 21417‐52) daily for 6 days. Then the unconditioned medium was replaced with endothelial cell medium, constituted by human endothelial serum‐free medium (hESFM; Gibco, Cat# 11111044) supplemented with 1% platelet‐poor human plasma derived serum (PDS; Sigma, Cat# P2918), 20 ng/ml human basic fibroblast growth factor (R&D Systems, Cat# 233‐FB) and 10 μM retinoic acid (Fujifilm Wako Pure Chemicals, Cat# 188‐01113). After two days, the cells were dissociated into single cells with Accutase and plated at a density of 250,000 cells/cm^2^ onto 8‐well tissue culture chamber slides (Thermo) or tissue culture dishes coated with collagen IV (80 μg/ml; Sigma, Cat# C5533) and fibronectin (20 μg/ml; Sigma, Cat# F2518). Twenty‐four hours after subculture, cells were washed twice with calcium and magnesium‐containing phosphate‐buffered saline (PBS) (Gibco, Cat# 14040133); after that the medium was replaced with hESFM containing 1% PDS.

### 2.4 Acetylated low‐density lipoprotein uptake assay

iVECs were incubated with 10 μg/ml acetylated low‐density lipoprotein (LDL) conjugated to Alexa Fluor 488 (Invitrogen, Cat# L23380) in hESFM for 4 hr at 37°C. The nuclei were stained with Hoechst 33342 (2.5 μM; Sigma, Cat# B2261) for 40 min. After incubation, the cells were washed twice with PBS and visualized immediately using a fluorescent microscope (Axioplan2; Zeiss) with Cooled CCD Camera (DP73; Olympus).

### 2.5 Tube formation assay

Tube formation assay was performed as previously described (Lippmann et al., 2012). Eight‐well chamber slide was coated with 100 μl of Matrigel for 1 hr at 37°C. iVECs were dissociated using Accutase, suspended in hESFM supplemented with 40 ng/ml vascular endothelial growth factor (VEGF) (R&D Systems, Cat# 293‐VE), and seeded at a density of 100,000 cells/cm^2^. After 12 hr incubation, the cells were observed using a phase‐contrast microscope (Zeiss).

### 2.6 Stereotactic ET‐1 injection and cell transplantation

ET‐1 injection was performed as previously described with some modifications (Ono, Imai, Miyawaki, Nakatomi, & Saito, 2016; Puentes et al., 2012). Rats were anesthetized with isoflurane (an effective, safe, and commonly used anesthetic) and placed in a stereotactic frame. Body temperature was maintained at 37°C using a homeothermic pad. The posterior limb of the left IC was the target for injection (2.0 mm posterior and 6.6 mm lateral from the bregma, 4.6 mm depth from the brain surface). The needle was angled away from the injection site at 30° to avoid potential damage to the hippocampal structures. ET‐1 solution (1 μl/injection, 100 pmol/μl; Peptide Institute, Cat# 4198‐s) with sterile PBS was injected into the targeted IC via a 10‐μl syringe (Hamilton) connected to a micropump (injection rate: 0.2 μl/min). After injection, the needle was left in place for 10 min to avoid backflow, and then it was slowly removed from the brain. We did not use analgesics for rats after surgery because we were worried that these compounds would affect the results. To minimize animal suffering, rats were allowed to recover for 2 hr in a 28°C room before returning to the home feeding room. Thirty‐two rats were prepared to establish the white matter infarct model. Twelve rats were excluded and discarded, however, because the size and location of the infarct were not controlled well, when analyzed using magnetic resonance imaging (described below).

Seven days after ET‐1 injection, when the white matter ischemic injury was established, 2 μl of the cell suspensions (human iVECs, human iPSCs, or rat MCs suspended in HBSS containing 0.2% bovine serum albumin (BSA) (0.2% BSA/HBSS) at the density of 50,000 cells/μl) or 0.2% BSA/HBSS was injected into each animal (five rats in each group) using the coordinates used for ET‐1 injection at a constant flow rate (0.2 μl/min).

### 2.7 Behavioral assessment

To assess the locomotor function, animals were evaluated using the footprint test as previously described (Puentes et al., 2012). On day 0, 7 and 21, the animals were placed on a narrow runway covered with a sheet of paper (1 m in length and 6 cm in width), after their hind limbs were stained with black ink (non‐toxic). The narrow runway ensured that the animals walked along a straight path. To improve the rat performance, a lamp was placed at the starting point, and a receptor dark cage was located at the endpoint to encourage animals to finish the task as fast as possible; when rats stopped during the evaluation, the data from non‐completed walking patterns were discarded, and the animals were returned to their home cages. The test was performed again 10 min later to get the completed walking patterns. All footprints were scanned and recorded by a scanner. For each animal, a series of eight sequential footprints (four right, four left, two cycles) were used to determine mean values of limb rotation (LR: the angle between a virtual line through the third digit and the center of the palm and a virtual line parallel to the walking direction).

### 2.8 Magnetic resonance imaging

Magnetic resonance (MR) imaging was carried out on day 7 after ET‐1 injection before cell transplantation or 0.2% BSA injection, and on day 21 using a small animal 1‐T benchtop MR scanner (Icon; Bruker Biospin). Rats were anesthetized with 5% isoflurane and maintained with 1.5% isoflurane. Respiration rates were monitored throughout the procedure, and body temperature was maintained at 37°C. T2‐weighted image was used to determine the precise lesion location: rapid‐ acquisition relaxation enhancement factor 5, repetition time 2,500 ms, echo time 60 ms with in‐plane resolution of 266 × 266 μm^2^, thickness 1,250 μm, and five slices.

### 2.9 Cryosection

Cryosection was performed as previously described with some modifications (Iijima et al., 2015). The animals were anesthetized with isoflurane and perfused transcardially with 4% paraformaldehyde (PFA) in PBS containing 10 U/ml heparin. The brains were immediately dissected and post‐fixed for 1 hr in the same fixative at 4°C, cryoprotected in 20% sucrose (Fujifilm Wako Pure Chemicals, Cat# 196‐00015) at 4°C for 2 days and then in 30% sucrose until equilibrated. Frozen coronal sections (20 μm in thickness, 120 serial slices per brain) with Tissue‐Tek O.C.T. Compound (Sakura Finetek) were cut using a cryostat (CM3050S; Leica Biosystems), mounted on pre‐coated glass slides (Matsunami), and placed at room temperature for 1 hr to air dry. The sections were stored at −80°C until staining.

### 2.10 Immunofluorescent staining

The cells and brain sections were fixed with 4% PFA solution for 10 min, incubated with 3% BSA in PBS containing 0.3% Triton X‐100 for 30 min, and then incubated with primary antibodies including mouse monoclonal antibodies directed against VE‐cadherin (1:100; Santa Cruz, Cat# sc‐9989, RRID: AB_2077957), CD31 (1:50; Abcam, Cat# ab24590, RRID: AB_448167), zonula occludens‐1 (ZO‐1) (1:100; ThermoFisher, Cat# 33‐9100, RRID: AB_2533147), Occludin (1:100; ThermoFisher, Cat# 33‐1500, RRID: AB_2533101), GLUT1 (1:200; ThermoFisher, Cat# SPM498, RRID: AB_1074665), STEM121 (1:500; Takara Bio, Cat# Y40410, RRID: AB_2801314), RECA‐1 (1:100; Bio‐Rad, Cat# MCA970GA, RRID: AB_567193), and rabbit polyclonal antibodies directed against CD31 (1:25; Lab Vision, Cat# RB‐10333, RRID: AB_720501), von Willebrand factor (vWF) (1:100; Santa Cruz, Cat# sc‐14014, RRID: AB_2241707), Claudin‐5 (1:100; ThermoFisher, Cat# 34‐1600, RRID: AB_2533157), NG2 (1:100; Millipore, Cat# AB5320, RRID: AB_91789), glial fibrillary acidic protein (GFAP) (No dilution; Dako, Cat# IR524), Olig2 (1:200; IBL, Cat# 18953, RRID: AB_494617) (Supplemental Table S1) at 4°C overnight. After washing with PBS, the samples were incubated with fluorophore‐conjugated secondary antibodies for 2 hr at room temperature. Nuclei were stained with Hoechst 33342 (1 μg/ml) for 1 hr at room temperature. For Olig2 immunostaining, antigen retrieval was performed at 120°C for 20 min in trisodium citrate buffer (10 mM, pH 6.0) before blocking non‐specific binding sites. For vWF, lacto‐*N*‐fucopentaose I (LNFP I) (1:2000; Funakoshi, Cat# FDV‐0014) and ED‐1 (1:5,000; Bio‐Rad, Cat# MCA341R, RRID: AB_2291300) (Supplemental Table S1) detection, the brain sections were immunostained using a TSA system (Perkin Elmer) to amplify the positive tyramide signal. The samples were observed using a fluorescent microscope (Axioplan2; Zeiss) with Cooled CCD Camera (DP73; Olympus).

### 2.11 Luxol fast blue staining

Brain sections were stained with luxol fast blue (LFB) as previously described (Shimauchi‐Ohtaki et al., 2019). To calculate the volume of the IC, 12 slices were stained across the injured zone (distance between slices: 200 μm) and the left and right IC areas were measured in each slice. Sections were visualized using a fluorescence microscope (BZ‐X710; Keyence).

### 2.12 Statistical analysis

All values are shown as mean ± standard error (SE). For analysis of footprint test, infarct area in MR images and damaged area in LFB staining, data were assessed with Shapiro–Wilk test for normality in each group and with Bartlett test for homogeneity of variances in different groups. Differences between groups were assessed with one‐way ANOVA followed by the post hoc Tukey–Kramer test. All statistical analysis was performed, then quantification graphs were generated by using GraphPad Prism 7.0 (GraphPad Prism Software). Statistical significance was inferred at *p* < .05. No sample size calculation was performed, and no blinding was performed. No exclusion criteria were pre‐determined and no outlier data were removed.

## 3 RESULTS

### 3.1 Highly purified iVECs were obtained from human iPSCs

**Figure 1:**
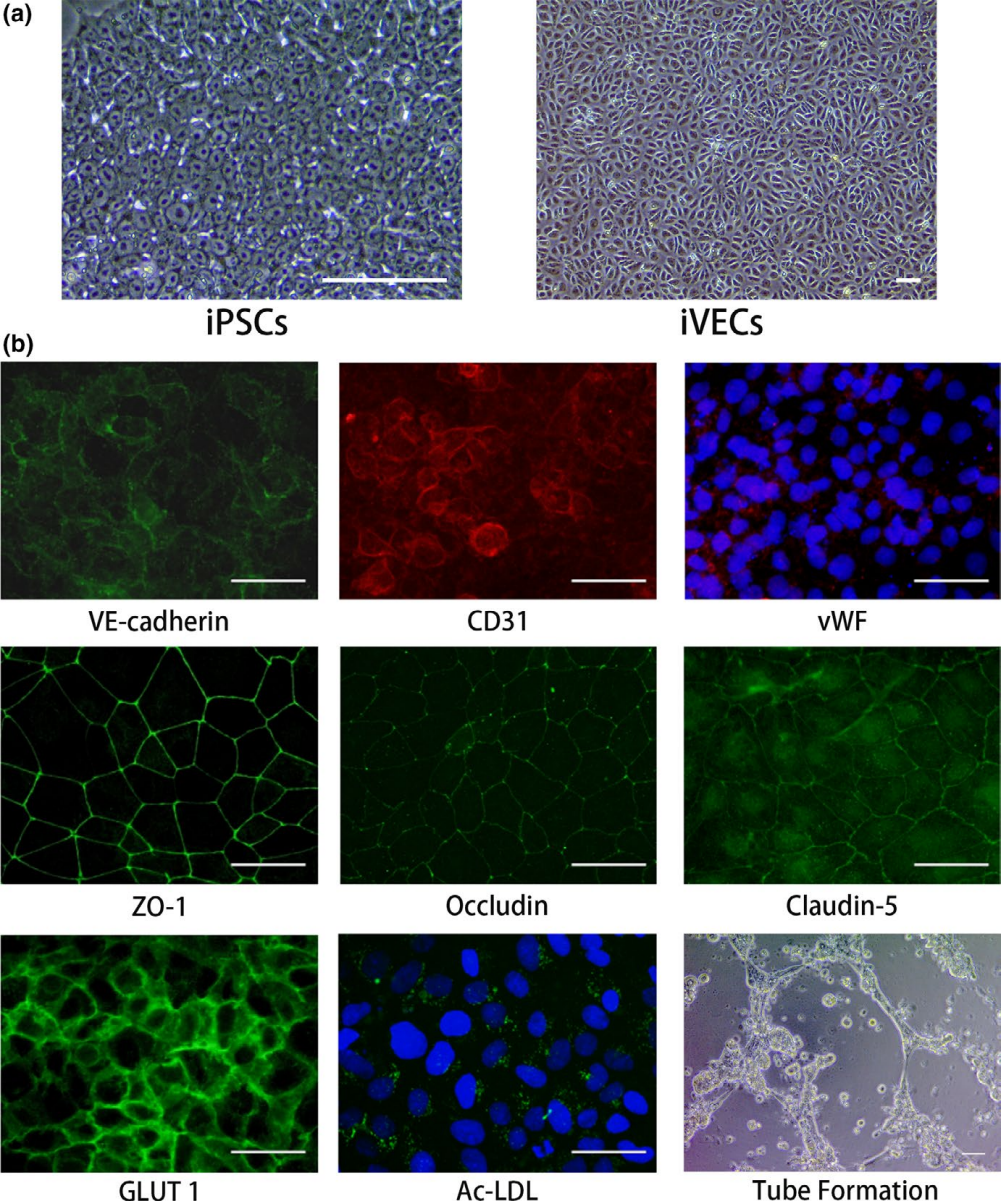
Differentiation of iVECs from human induced pluripotent stem cells (iPSCs) for transplantation. (a) Phase‐contrast images show morphology of iPSCs and differentiated iVECs. Scale bars: 100 μm. (b) Expression of endothelial markers (VE‐cadherin, CD31, von Willebrand factor [vWF]), tight junction proteins (ZO‐1, occludin, claudin‐5), and glucose transporter (GLUT1). iVECs took up fluorescent acetylated low‐density lipoprotein, and lead to tube formation with 40 ng/ml vascular endothelial growth factor. Nuclei (blue) were stained by Hoechst 33342. Scale bars: 50 μm

To obtain vascular endothelial cells for transplantation, human iPSCs were differentiated as described previously (Kokubu et al., 2017; Lippmann et al., 2014, 2012; Stebbins et al., 2016). Compared to undifferentiated iPSCs, the differentiated cells displayed characteristic endothelial cell morphology and cobblestone‐like arrangement (Figure 1a). Immunocytochemical staining showed that almost all the cells expressed endothelial markers: VE‐cadherin, CD31, and vWF (Figure 1b). They also expressed glucose transporter GLUT1 and tight junction proteins: ZO‐1, occludin, and claudin‐5 (Figure 1b). The cells exhibited the ability to uptake acetylated LDL (Ac‐LDL), one of the characteristics of vascular endothelial cells (Figure 1b). In addition, these cells showed tube formation in the presence of VEGF (Figure 1b). These results confirmed that highly purified iVECs were obtained for transplantation.

The majority of MCs prepared for transplantation as control were positive for NG2 by immunocytochemistry (Figure S1a). None of these cells were positive for endothelial markers such as CD31 (Figure S1b) or vWF (Figure S1c).

### 3.2 iVEC transplantation promoted behavioral recovery of motor deficits

**Figure 2:**
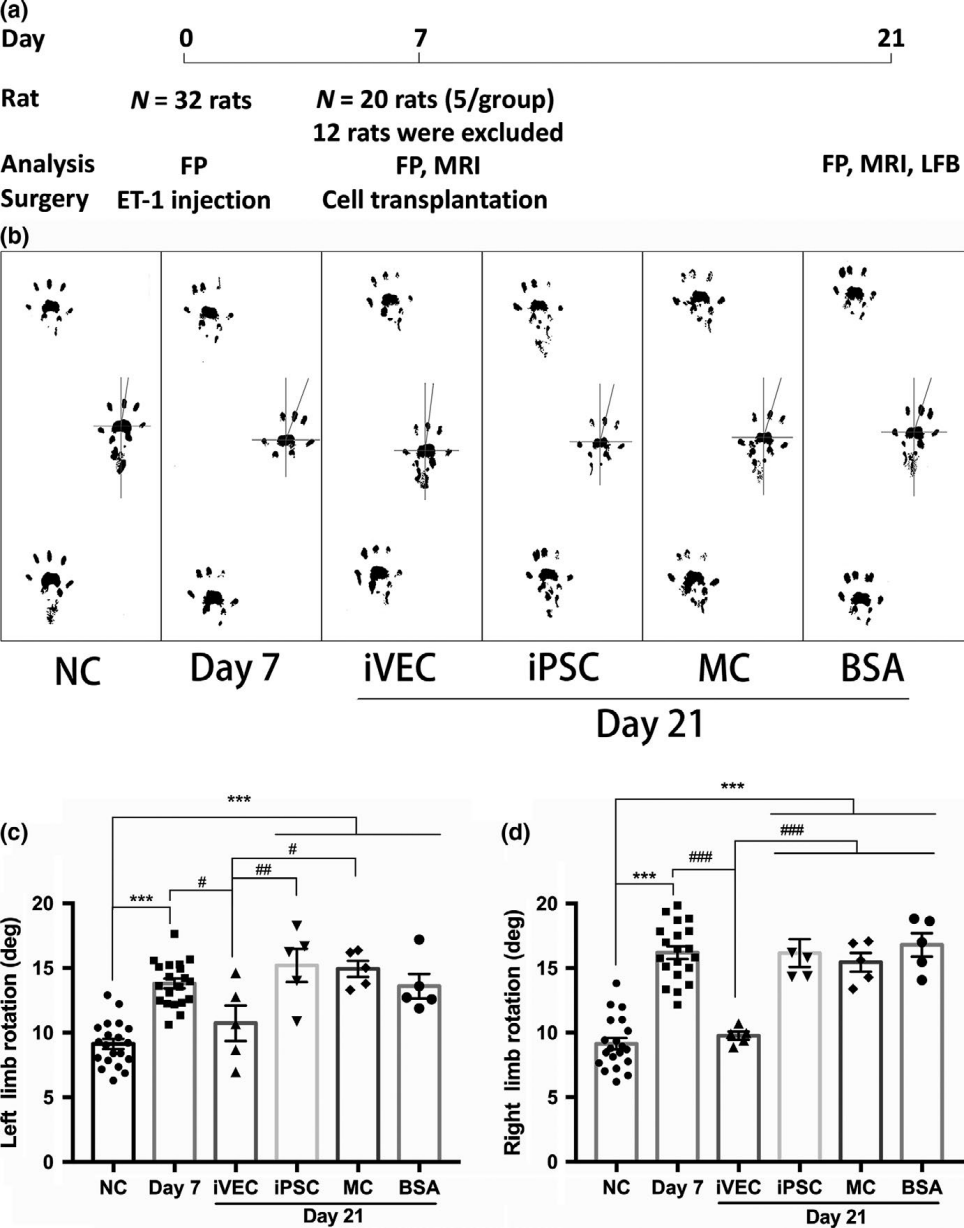
Transplanted iVECs improved the behavioral outcome. (a) The timeline for the experimental procedure in this study is shown. endothelin‐1 was injected into the left IC to induce infarction on day 0. iVEC, induced pluripotent stem cell (iPSC), meningeal cell (MC), or bovine serum albumin (BSA) were injected into each animal using the initial coordinates on day 7. Footprint (FP) test was performed to evaluate the locomotor function on day 0, day 7, and day 21. Hind limb footprints were registered and limb rotation angle was measured. Infarct volume was evaluated by magnetic resonance imaging (MRI) on day 7 and day 21. All rats were killed for histochemical analysis, such as luxol fast blue (LFB) staining, on day 21. (b) Representative tracks were registered on day 0, day 7, and day 21. (c, d) Statistical analysis of the relationship between each group. All data are expressed as mean ± SE. *N* = 20 rats in normal control (NC) group and day 7 group; *N* = 5 rats in iVEC, iPSC, MC, and BSA groups. ***p < .001 against normal control group. ^#^ *p* < .05, ^##^ *p* < .01, and ^###^ *p* < .001 against iVEC group

Ischemic white matter infarct was induced by ET‐1 injection into the left IC of rat brain on day 0. To evaluate the change of behavioral performance induced by ischemic damage, footprint test was performed, and hind limb rotation angle was measured from the footprints (Puentes et al., 2012). Footprint test was performed before ET‐1 injection at day 0. It was performed again 7 days after ET‐1 injection. MR imaging was carried out after footprint test and the cells were transplanted into the site of ischemic demyelinating lesion. On day 21, after footprint test and MR examination, all rats were killed under deep anesthesia with isoflurane for histochemical analysis, such as LFB staining (Figure 2a).

Induction of ischemic damage by ET‐1 injection significantly increased both left and right LR angles on day 7, when compared to those of rats in untreated normal control group (Figure 2). This result suggests that ET‐1 injection caused enough damage to white matter to induce motor disabilities. The increase in LR angle of both hind limbs may be because of the adoption of compensatory exercise strategies during walking (Farr, Liu, Colwell, Whishaw, & Metz, 2006; Puentes et al., 2012).

After footprint test on day 7, rats were separated into four groups. To each of three groups, three different types of cells (iVECs, iPSCs, MCs) were transplanted into the infarct area of rat brains. To one group, as control, only 0.2% BSA/HBSS was injected into the infarct area. Two weeks later, on day 21, footprint test was performed again to evaluate effects on the behavioral outcome by cell transplantation. On day 21, human iVEC transplantation remarkably decreased LR angle of both hind limbs when compared to that on day 7, and there was no significant difference in LR angles between iVEC‐transplanted rats and normal control rats. In contrast, the LR angles of animals treated with human iPSC or rat MC transplantation or BSA injection were not reduced compared with those on day 7 (Figure 2). These data indicated that iVEC transplantation improved behavioral outcome and recovered the motor disturbances caused by ischemic white matter infarct.

### 3.3 iVEC transplantation reduced the area of ischemic white matter infarct

**Figure 3:**
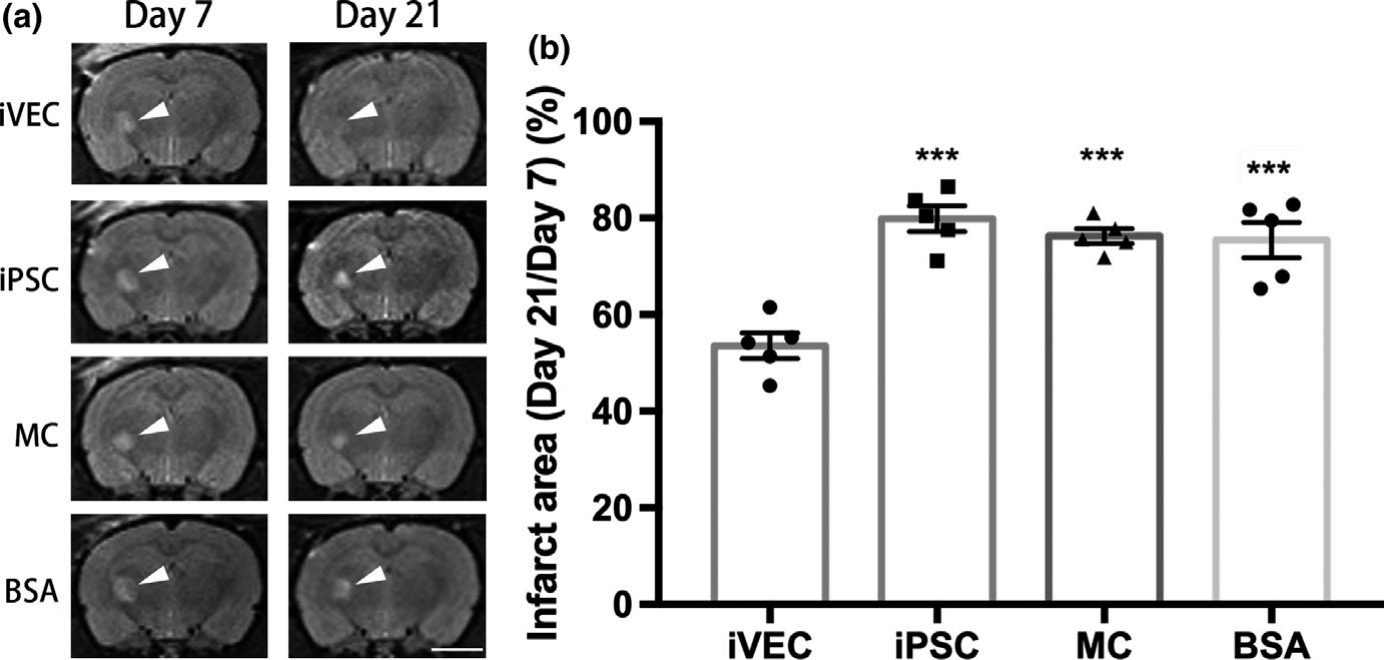
iVEC transplantation reduced white matter infarct on day 21. (a) T2‐weighted magnetic resonance (MR) images of rat brains with ischemic infarct (arrowheads) in the IC induced by endothelin‐1 injection on day 7 and the same rat brains on day 21 (14 days after cell transplantation). Scale bar: 5mm. (b) Quantification of the percentage infarct area in IC on day 21 relative to that on day 7. All data are expressed as mean ± SE. *N* = 5 rats in each group. ***p < .001 against iVEC group

To evaluate changes in infarction area induced by ET‐1 injection and affected by cell transplantation, T2‐weighted MR images of rat brains were captured on day 7 and day 21 in the same rats. Seven days after ET‐1 injection (day 7), a hyperintense area was found in the ET‐1‐injected IC, indicating that the model of white matter infarct in rat brain was successfully established (Figure 3a). Two weeks after various cell transplantations or BSA injection (day 21), the infarct size was examined again. After that, relative infarct area (day 21/day 7) was evaluated as an index to reflect white matter damage. Although the relative infarct area treated with human iPSC and rat MC transplantation was reduced to about 20%, there was no significant difference between these two groups and the 0.2% BSA‐injected group (Figure 3a,b). These data indicate that human iPSC or rat MC transplantation did not improve ET‐1‐induced white matter infarct. The relative infarct area in human iVEC‐transplanted group, however, was dramatically reduced when compared with the other groups (Figure 3b). These results demonstrate that iVEC transplantation attenuated ischemic white matter damage.

### 3.4 iVEC transplantation promoted remyelination in white matter infarct

**Figure 4:**
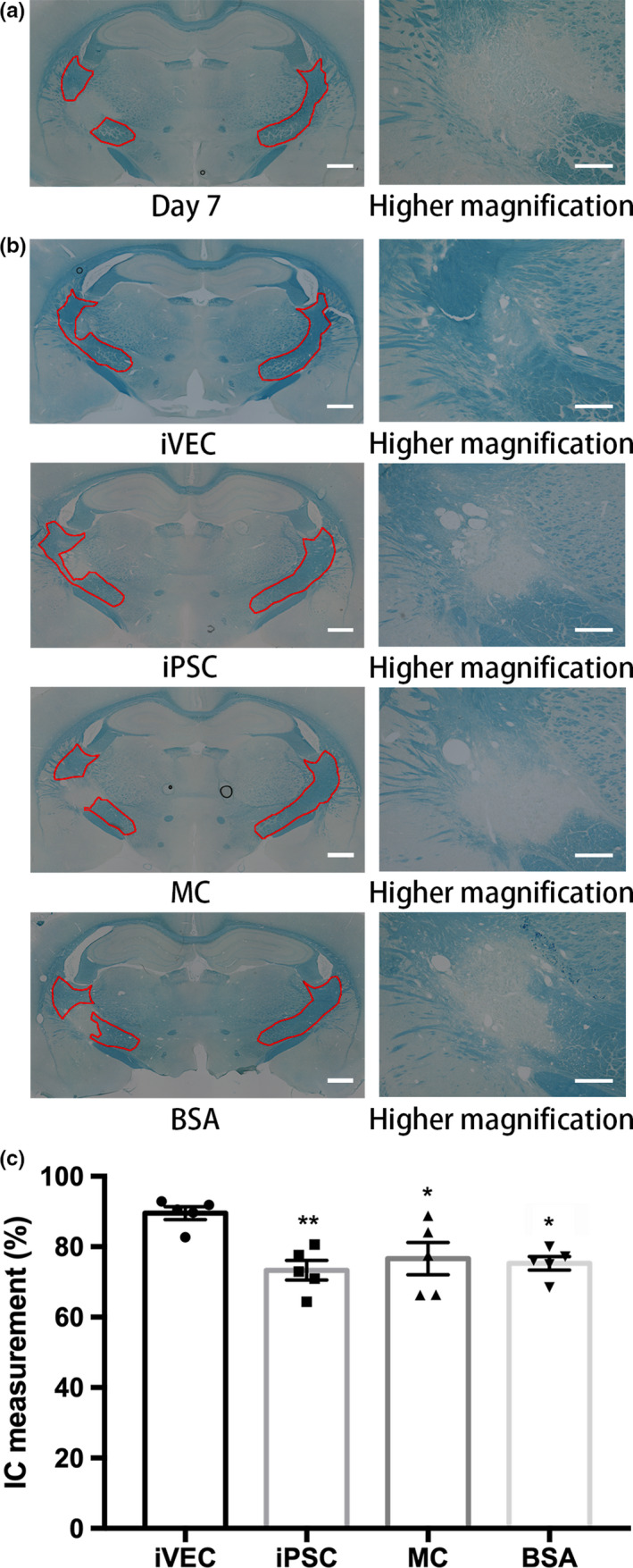
iVEC transplantation improved remyelination in ischemic white matter lesion. To quantify remyelination in the injured IC, the volume of the myelinated IC was measured using serial brain sections stained with luxol fast blue (LFB). (a) LFB staining image of white matter injury after 7 days endothelin‐1 injection. Demyelinated lesion in the rat brain section was evident under higher magnification (right). (b) Typical LFB staining images of rat brain sections on day 21 in each group. Scale bars: 500 μm (left column) and 200 μm (right column). (c) Quantification of damaged IC on day 21. All data are expressed as mean ± SE. *N* = 5 rats in each group. *p < .05 and **p < .01 against iVEC group

Myelin was stained with LFB to assess the demyelinating lesion. On day 7, histological observation showed that the ET‐1‐injected area was not stained with LFB (Figure 4a). The boundary of demyelination was evident under higher magnification and the broad area in the IC was demyelinated (Figure 4a). These data indicated that the myelin sheath was damaged in ET‐1‐injected IC and establishment of white matter infarct model was confirmed. On day 21, the infarct lesion was reduced and remyelination was recognized in ET‐1‐treated IC of iVEC‐transplanted rats, compared with the BSA‐injected group (Figure 4b). Neither human iPSC nor rat MC transplantation, however, showed this effect (Figure 4b). To quantify remyelination in the injured IC, the volume of the myelinated IC was measured using serial brain sections stained with LFB. The analysis revealed that the iVEC‐transplanted rats made a significant recovery in IC volume, compared with those in other groups (Figure 4c). These data suggested that iVEC transplantation improved remyelination in the injured white matter and contributed to the recovery of IC.

### 3.5 Transplanted iVECs survived and retained their endothelial characteristics for two weeks in rat brain, while transplanted iPSCs survived and began to exit the undifferentiated state under this condition

**Figure 5:**
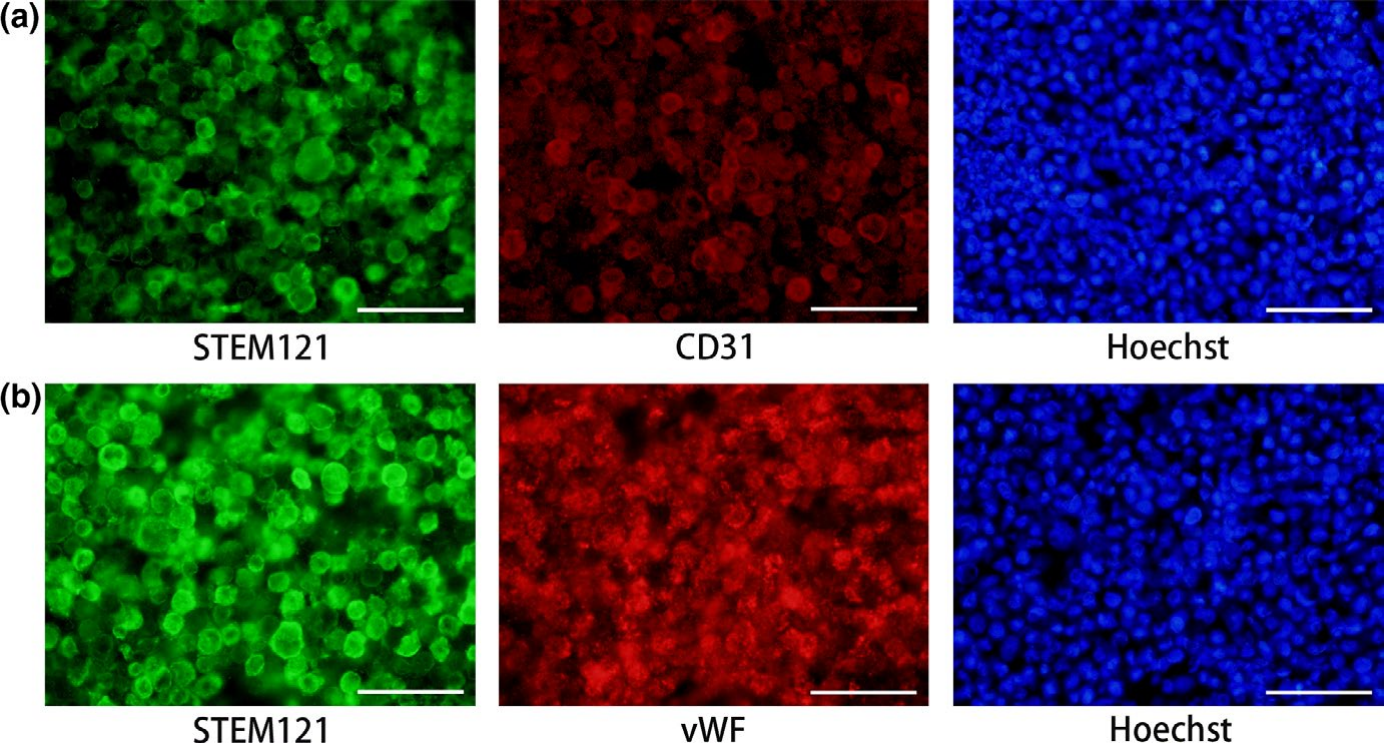
iPSC‐derived vascular endothelial cells (iVECs) survived for 14 days after transplantation. Transplanted iVECs which expressed a human‐derived cell marker (STEM121) also expressed endothelial markers, CD31 (a) and von Willebrand factor (vWF) (b). Nuclei (blue) were stained by Hoechst 33342. Scale bars: 50 μm

Double immunohistochemical staining for a human cytoplasm specific antibody (STEM121) and endothelial cell markers (CD31 or vWF) revealed that STEM121‐positive cells expressed endothelial markers, indicating that transplanted iVECs survived and maintained the characteristics of endothelial cells for 2 weeks after transplantation (Figure 5).

Transplanted iVECs were labeled with a human CD31 antibody, which does not label rat endothelial cells. On the other hand, rat blood vessels were stained by a RECA‐1 antibody, which reacts only with the rat endothelial cell antigen. Double immunostaining for human CD31 and RECA‐1 showed that no human CD31‐positive cells were integrated into the host vasculatures (Figure S2). This result indicates that the beneficial effects of transplanted iVECs on ischemic demyelinating injury are not because of promotion of angiogenesis in the infarct area, confirming our previous result (Puentes et al., 2012).

To examine whether engrafted iPSCs survived and remained undifferentiated for 2 weeks in rat brain, we used an antibody (R‐17F) against LNFP I, a marker for stemness of human iPSCs. Immunostaining with STEM121 and anti‐LNFP I (R‐17F) antibodies revealed that transplanted human iPSCs survived and remained undifferentiated 1 day after transplantation (Figure S3a). Two weeks after transplantation, STEM121‐positive iPSCs survived and still existed in the transplanted area of rat brain. The signal of LNFP I in STEM121‐positive cells, however, was reduced (Figure S3b), suggesting that transplanted iPSCs began to exit the undifferentiated state and differentiate into some cell type(s).

### 3.6 iVEC transplantation increased oligodendrocyte lineage cells, suppressed inflammation, and inhibited reactive astrocytogenesis in ischemic demyelinating lesion

**Figure 6:**
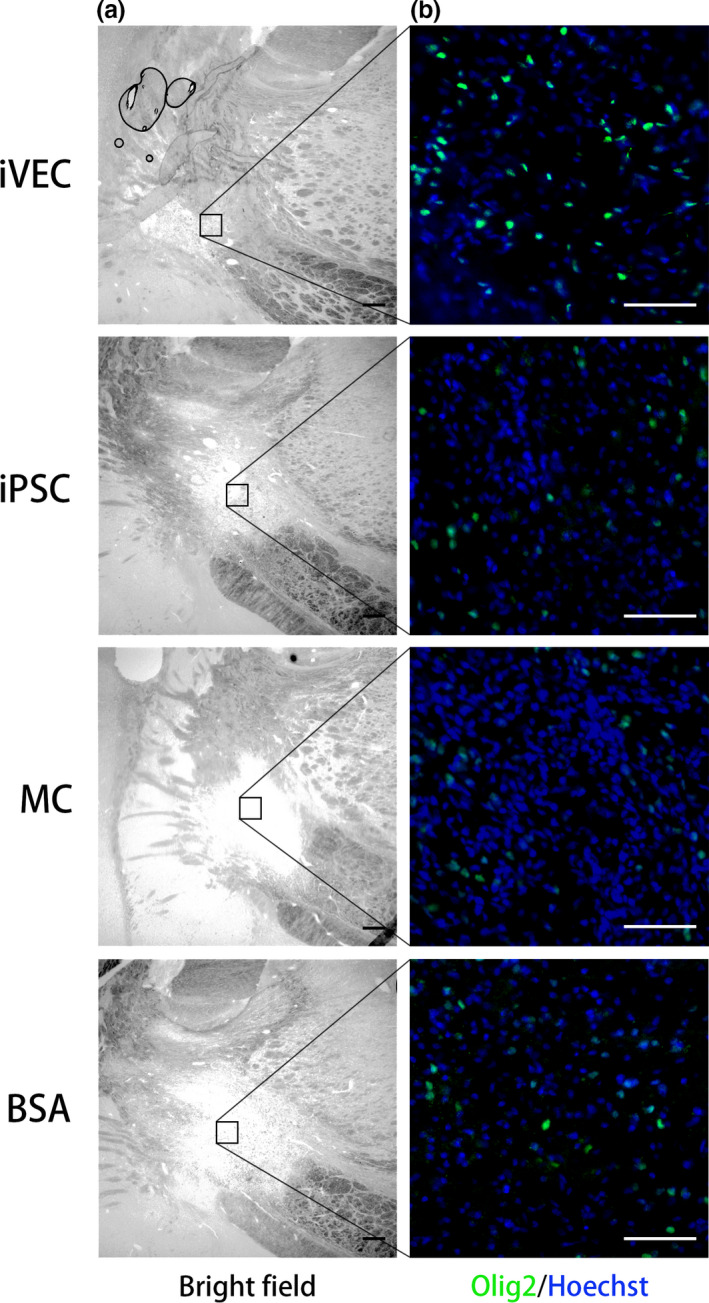
iPSC‐derived vascular endothelial cell (iVEC) transplantation increased oligodendrocyte (OL) lineage cells in endothelin‐1‐injected internal capsule. (a) Representative bright field images of the infarct lesion in each group on day 21. Scale bars: 200 μm. (b) Higher magnification images of the insets in (a). OL lineage cells were stained by an Olig2 antibody. Nuclei (blue) were stained by Hoechst 33342. Scale bars: 50 μm

Myelin sheaths are produced by mature OL differentiated from OPCs. To evaluate whether cell transplantation affects OL lineage cells, they were stained by an Olig2 antibody. Fourteen days after transplantation, our results showed that the density of Olig2‐positive cells was significantly increased in ischemic demyelinating lesion of iVEC‐transplanted rats, compared with human iPSC‐ or rat MC‐transplanted, or BSA‐injected animals (Figure 6).

**Figure 7:**
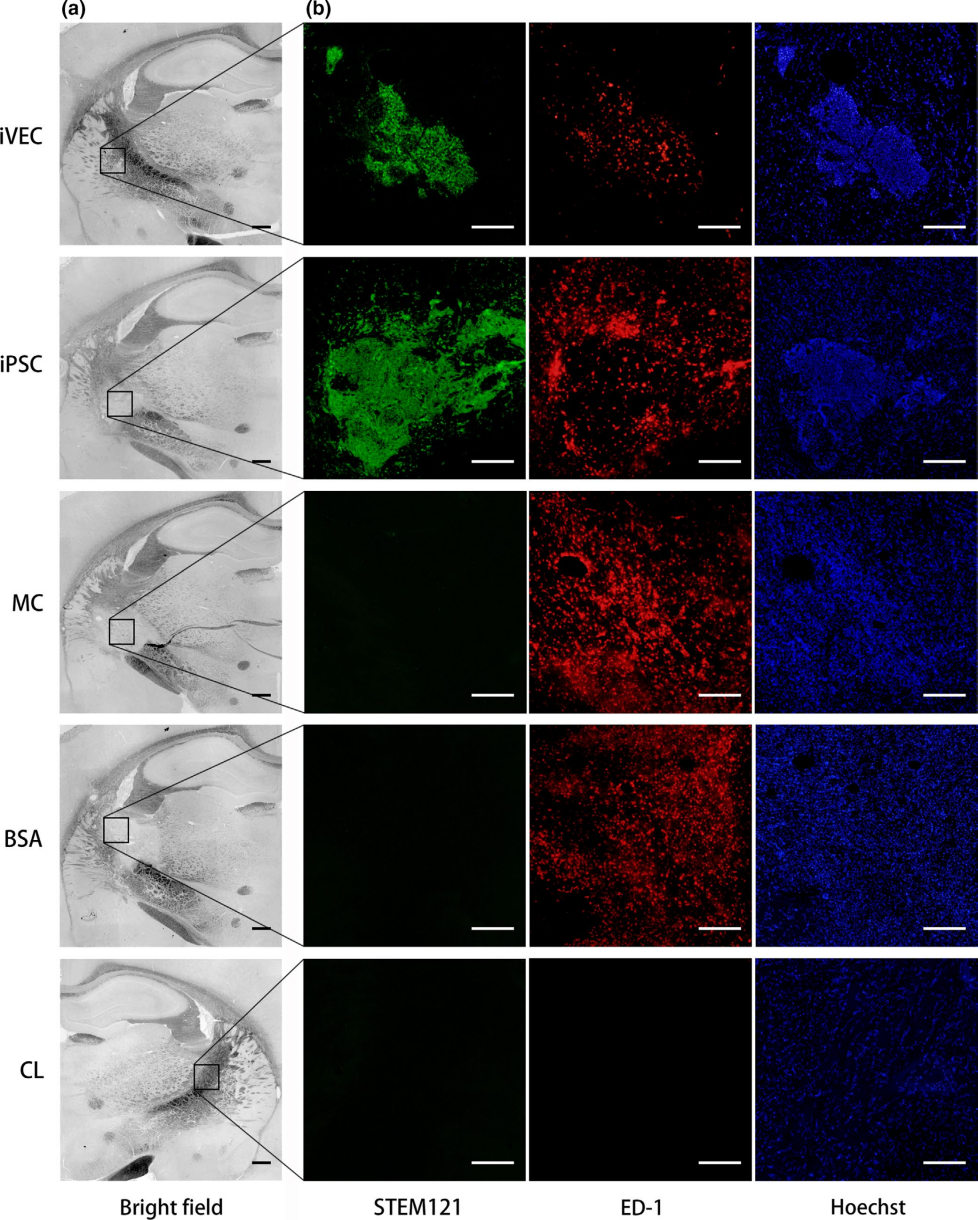
iPSC‐derived vascular endothelial cell (iVEC) transplantation suppressed inflammatory response in ischemic demyelinating lesion. (a) Representative bright field images of the infarct lesion in each group on day 21. Scale bars: 500 μm. (b) Higher magnification images of the insets in (a). A low concentration of an ED‐1 antibody was used to stain activated microglia with signal amplification by a TSA system. Then the cells were labeled with a STEM121 antibody. Nuclei (blue) were stained by Hoechst 33342. Scale bars: 200 μm

Inflammation was evaluated by immunostaining using an ED‐1 antibody, which labels activated microglia and macrophages. Transplanted human cells were labeled by a STEM121 antibody. On day 21, increased ED‐1‐positive cells were observed in the ischemic demyelinating lesion. No signal was observed in the contralateral side (CL). Transplanted iVECs significantly reduced the ED‐1‐positive cells compared with other groups, indicating that iVEC transplantation suppressed the inflammatory response in ET‐1 injured region (Figure 7).

**Figure 8:**
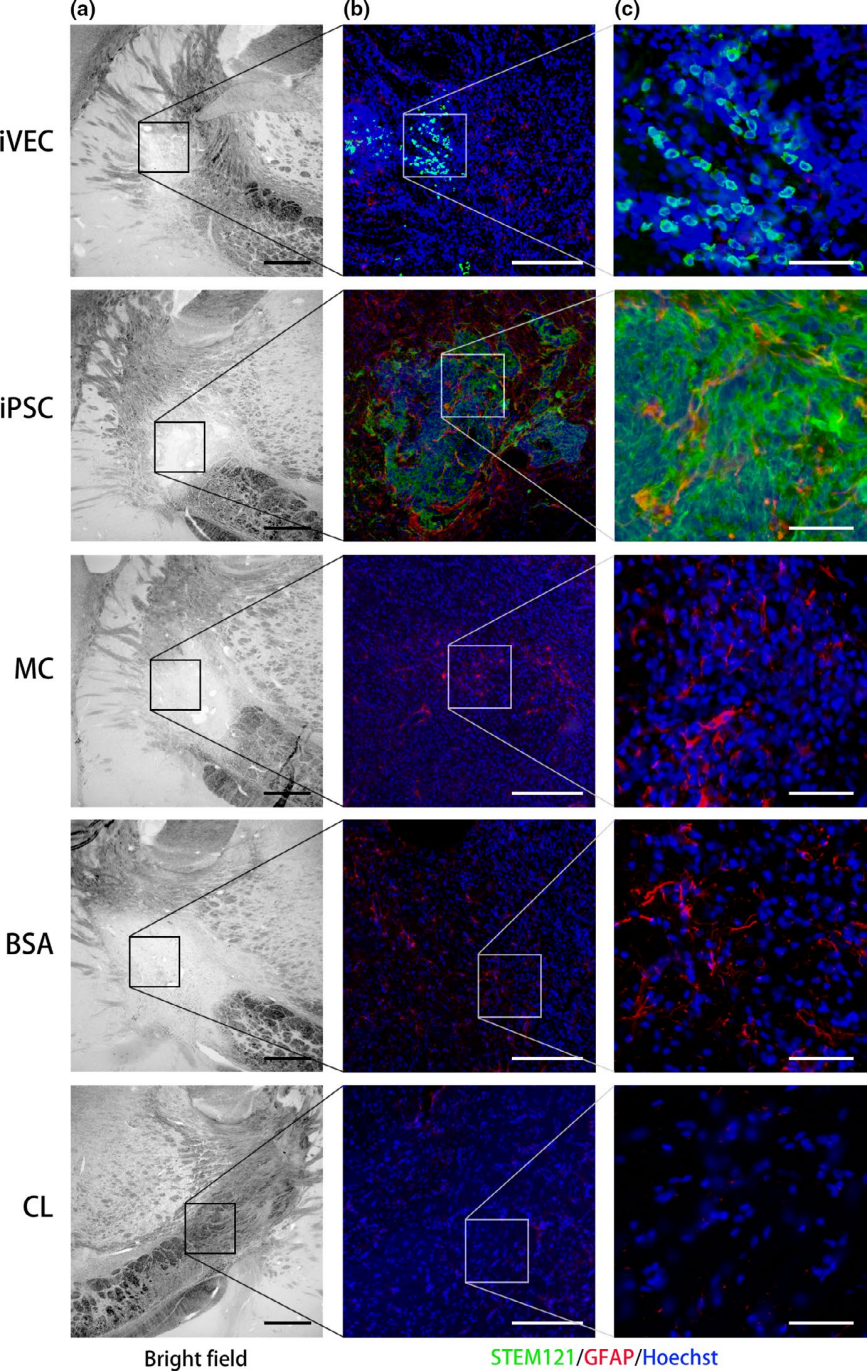
iPSC‐derived vascular endothelial cell (iVEC) transplantation decreased reactive astrocytes in the infarct lesion. (a) Representative bright field images of the infarct lesion in each group on day 21. (b) Higher magnification images of the insets in (a). (c) Higher magnification images of the insets in (b). Astrocytes were labeled by a glial fibrillary acidic protein (GFAP) antibody and transplanted human cells were labeled by a STEM121 antibody. Nuclei (blue) were stained by Hoechst 33342. Scale bars: 200 μm (left and middle column) and 50 μm (right column)

After injury, astrocytes in and around the injured region increase their volume and expression of GFAP. These cells are called reactive astrocytes (for review, see Escartin, Guillemaud, Carrillo‐de, & A., 2019). We stained the sections of ET‐1‐injured brains to detect reactive astrocytes in ischemic demyelinating lesion on day 21 (Figure 8). No signal of GFAP was observed in the intact IC (CL in Figure 8). By contrast, reactive astrocytes appeared in the ET‐1‐induced ischemic demyelinating area (iPSC, MC, BSA in Figure 8). Transplantation of iVECs, however, significantly decreased the number of GFAP‐positive cells in demyelinating lesion (iVEC in Figure 8).

## 4 DISCUSSION

We previously reported that transplantation of MVECs prepared from rat brain improved the behavioral outcome and promoted remyelination in the rat model of white matter infarct (Puentes et al., 2012). The major obstacles for clinical application of these findings are the difficulty of obtaining a certain amount of human vascular endothelial cells and the problem of xenograft rejection (Anderson et al., 2011). In this study, we found that transplantation of iVECs recovered locomotor deficit, reduced infarct volume, and promoted remyelination, thus ameliorating ischemic stroke in the rat model of white matter infarct. Employment of iVECs for treatment of white matter infarct can circumvent the obstacles described above. First, a desirable amount of human vascular endothelial cells can be obtained from the iPS cell stock. Second, iVEC transplantation is expected to evoke no xenograft rejection.

We tried several ways of differentiating iPSCs into vascular endothelial cells. Among them we adopted the method established by Lippmann and others (Lippmann et al., 2014, 2012). The cells obtained by this method have been reported to express endothelium‐specific mRNAs, such as the endothelial cell‐specific adhesion molecule ESAM (Delsing et al., 2018) and several brain endothelium‐specific transporters (Lippmann et al., 2012). The endothelial cells we obtained (iVECs) displayed characteristic endothelial cell morphology and exhibited the ability to uptake Ac‐LDL, one of the characteristics of vascular endothelial cells (Figure 1). Although it was reported that the cells obtained by this method expressed low levels of proteins and mRNA for CD31, vWF, and VE‐cadherin (Delsing et al., 2018), almost all iVECs expressed endothelial markers, such as VE‐cadherin, CD31, vWF, and tight junction proteins (ZO‐1, occludin, claudin‐5), as shown in Figure 1b. The discrepancy between the previous report and ours remains undetermined. One possibility for this discrepancy is the difference in iPS cells used in their studies and ours. To further confirm that iVECs are *bona fide* endothelial cells, tube formation assay was performed. These cells exhibited the ability to form tubes in the presence of VEGF (Figure 1b). Recently Lippmann's group developed the method to shorten differentiation time (Hollmann et al., 2017). Further study is needed to develop the iPS differentiation technology with shorter time, simpler process, and higher purity.

We transplanted iVECs, rat MCs, and human iPSCs into the site of demyelination 7 days after induction of the infarct. Among the cells transplanted, only iVECs showed beneficial effects on white matter infarct (Figures 2, 3, 4, 6, 7 and 8). Double immunostaining with a human cytoplasm specific antibody, STEM121, and antibodies against endothelial markers revealed the survival of iVECs and their maintenance of endothelial characteristics 2 weeks after transplantation (Figure 5). Rat MCs were previously shown to survive for 2 weeks after transplantation (Puentes et al., 2012), and human iPSCs were also shown in this study to survive for the same period (Figure S3). Thus, it is suggested that maintenance of endothelial characteristics is crucial to the therapeutic effect of iVEC transplantation on white matter infarct.

Although xenograft rejection is a major obstacle to studying human‐derived cells in preclinical animal models (Beldick et al., 2018), we did not observe any evidence of immune rejection in human iPSC or iVEC‐transplanted rats. Some studies have reported the success of transplantation of human neural stem cells into rat brains (Daadi et al., 2010; Jeong et al., 2003; Ji et al., 2015; Zalfa et al., 2019). These results may be explained by the brain being an immune‐privileged site for transplantation, enabling xenograft to survive in the brain for extended periods of time without immune rejection (Barker & Billingham, 1977). There is also a possibility that human iPSCs and iVECs have a similar immunomodulatory function as that of bone marrow mesenchymal stem cells in the host brain (Mohamad et al., 2013). Several articles have reported that transplanted human iPSCs or iPSC‐induced functional cells survived in animal brains (Chau et al., 2014; Kawai et al., 2010; Lam, Lowry, Carmichael, & Segura, 2014; Wang et al., 2013). In the case of clinical application, however, utmost care must be taken to avoid xenograft rejection, as all the reports showing no rejection have been on human cells transplanted in animal brains. In this regard, employment of iVECs in therapy for white matter infarct seems much safer compared to that of non‐human cells.

Immunofluorescence staining for STEM121 showed that transplanted iPSCs survived for 14 days after transplantation (Figure S3b). The expression level of LNFP I, a marker for stemness, in these cells was much lower after 14 days, compared to that in the cells 1 day after transplantation, indicating that transplanted iPSCs had exited the undifferentiated state within 14 days (compare Figure S3a,b). In this study, we did not examine which type(s) of iPSCs differentiated. Kawai and others reported that transplanted iPSCs led to the formation of tridermal teratomas which delayed the recovery of stroke (Kawai et al., 2010). In this study, iPSC transplantation neither worsened behavioral outcome nor increased infarct volume during the period we observed. An extreme care must be taken, however, to employ highly purified iVEC preparation, without contamination by undifferentiated iPSCs, for treatment of white matter infarct.

We previously reported that MVEC transplantation increased the number of OPCs by inhibiting apoptotic death in the rat model of white matter infarct (Iijima et al., 2015). We also reported that EVs secreted from endothelial cells promoted survival, proliferation and motility of cultured OPCs (Kurachi et al., 2016). It seems plausible that iVECs exert beneficial effects on white matter infarct just in the same way as MVECs do. In this study, it was revealed that iVEC transplantation represses both immune response and reactive astrocytogenesis. Further study is required to reveal the relative contribution of repression of immune response and/or reactive astrocytogenesis to the beneficial effects of iVEC transplantation. We have been trying to identify the beneficial molecules in the EVs prepared from the conditioned medium of cultured MVECs. The progress has been slow so far unfortunately, however, because the quantity of EVs prepared from primary cultured MVECs is too small for biochemical analysis. It is easier to obtain iVECs from iPSCs, compared to obtaining primary culture of MVECs, in the amount sufficient for preparation of EVs for biochemical analysis. We are now planning to analyze the proteins and microRNAs contained in the EVs from iVECs. Identification of the molecules in the EVs responsible for the beneficial effects on white matter infarct, together with the direct employment of iVECs, would prove powerful for establishment of effective therapies for not only ischemic stroke but also for other demyelinating diseases.

## CONFLICT OF INTEREST

The authors declare no conflict of interest.
