# Supplementary material for: Transplantation of iPS‐derived vascular endothelial cells improves white matter ischemic damage
Source: J Neurochem. 2020 Jan 21;153(6):e14949. doi: 10.1111/jnc.14949 (PMC7317957; doi:10.1111/jnc.14949)

Transplantation of iPS-derived vascular endothelial cells improves white matter ischemic damage

Supplementary Information

Bin Xu, Masashi Kurachi, Hiroya Shimauchi-Ohtaki, Yuhei Yoshimoto, and Yasuki Ishizaki*

* Corresponding author:

Yasuki Ishizaki, MD, PhD, Department of Molecular and Cellular Neurobiology, Gunma University Graduate School of Medicine, 3-39-22 Showa-machi, Maebashi, Gunma 371-8511 Japan. E-mail: yasukiishizaki@gunma-u.ac.jp

Supplementary Table S1 The information for each primary antibody used in the present work.

Supplementary Figure S1 Characterization of primary cultured meningeal cells (MCs). Immunocytochemical staining of MCs showed that most of MCs were positive for NG2 (A), while none of them were positive for endothelial cell markers: CD31 (B) or vWF (C). Nuclei (blue) were stained by Hoechst 33342. Scale bars: 50 μm.

Supplementary Figure S2 Transplanted iVECs were not incorporated into the rat vasculatures. Brain sections were stained with a human CD31 antibody, which labels only human endothelial cells, and a RECA-1 antibody, which only reacts with the rat endothelial cell antigen. Double immunostaining for human CD31 and RECA-1 showed that no transplanted iVECs were incorporated into the host vasculatures. Nuclei (blue) were stained by Hoechst 33342. Scale bars: 50 μm.

Supplementary Figure S3 Transplanted iPSCs survived for 14 days and exited the undifferentiated state. A low concentration of an anti-LNFP I antibody (R-17F) was used to stain transplanted human iPSCs with signal amplification by TSA system. Then the cells were labeled with a STEM121 antibody. (A) Transplanted iPSCs were positive for both STEM121 and LNFP I on 1 day after transplantation. (B) Two weeks after transplantation, STEM121-positive cells were negative for LNFP I. (C) As control, the same concentration of the anti-LNFP I antibody was used without TSA system. No signal was detected when observed with the same exposure time as with (B). Nuclei (blue) were stained by Hoechst 33342. Scale bars: 200 μm (left column in A, B, C) and 50 μm (right column in A, B, C).

Supplementary Table S1 The information for each antibody used in the present work

Antibody	Supplier	Catalog Number	Host species	RRID	Dilution
VE-cadherin	Santa Cruz	sc-9989	Ms	AB_2077957	1:100
CD31	Lab Vision	RB-10333	Rb	AB_720501	1:25
	Abcam	ab24590	Ms	AB_448167	1:50
vWF	Santa Cruz	sc-14014	Rb	AB_2241707	1:100
ZO-1	ThermoFisher	33-9100	Ms	AB_2533147	1:100
Occludin	ThermoFisher	33-1500	Ms	AB_2533101	1:100
Claudin-5	ThermoFisher	34-1600	Rb	AB_2533157	1:100
GLUT1	ThermoFisher	SPM498	Ms	AB_1074665	1:200
STEM121	Takara Bio	Y40410	Ms	AB_2801314	1:500
NG2	Millipore	AB5320	Rb	AB_91789	1:100
ED-1	Bio-Rad	MCA341R	Ms	AB_2291300	1:5000
GFAP	Dako	IR524	Rb	–	–
RECA-1	Bio-Rad	MCA970GA	Ms	AB_567193	1:100
Olig2	IBL	18953	Rb	AB_494617	1:200
LNFP I (R-17F)	Funakoshi	FDV-0014	Ms	–	1:2000

Supplementary Figure S1

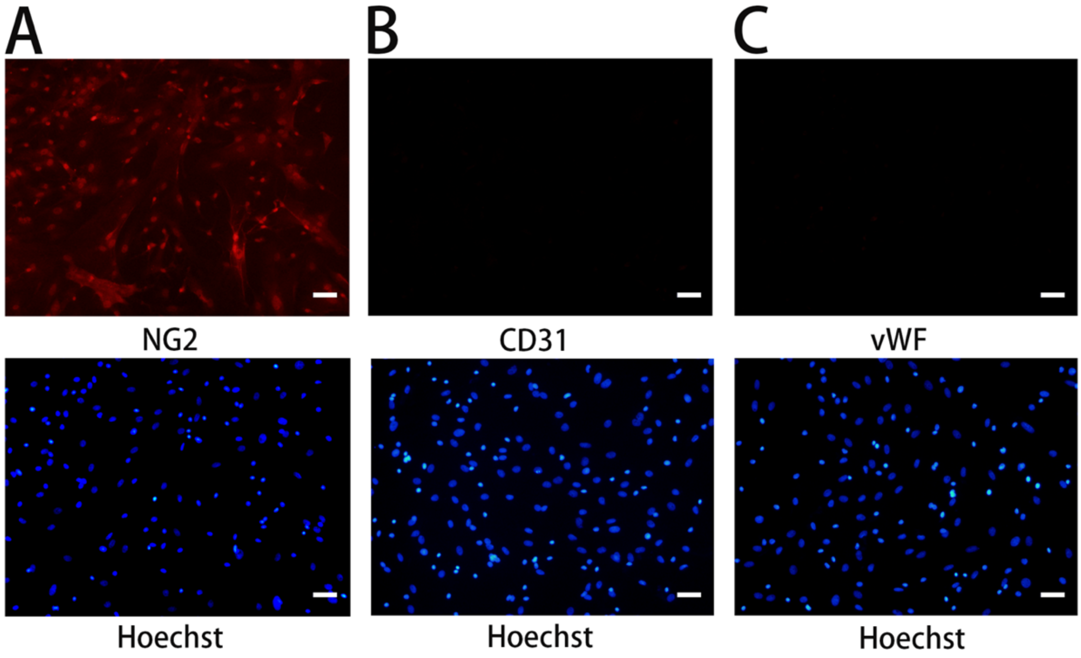

Supplementary Figure S2

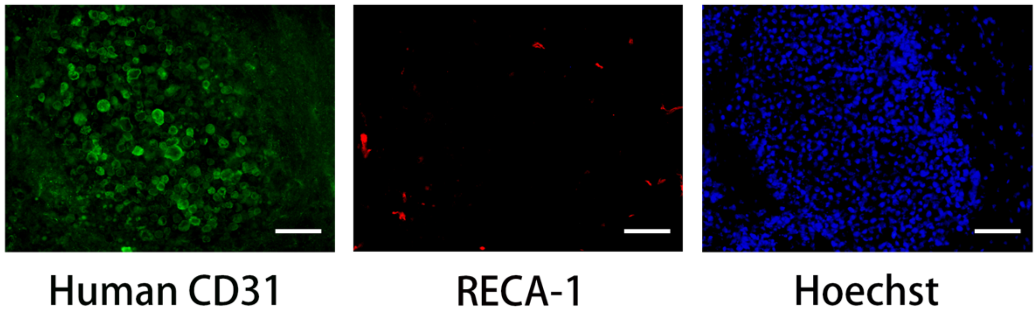

Supplementary Figure S3

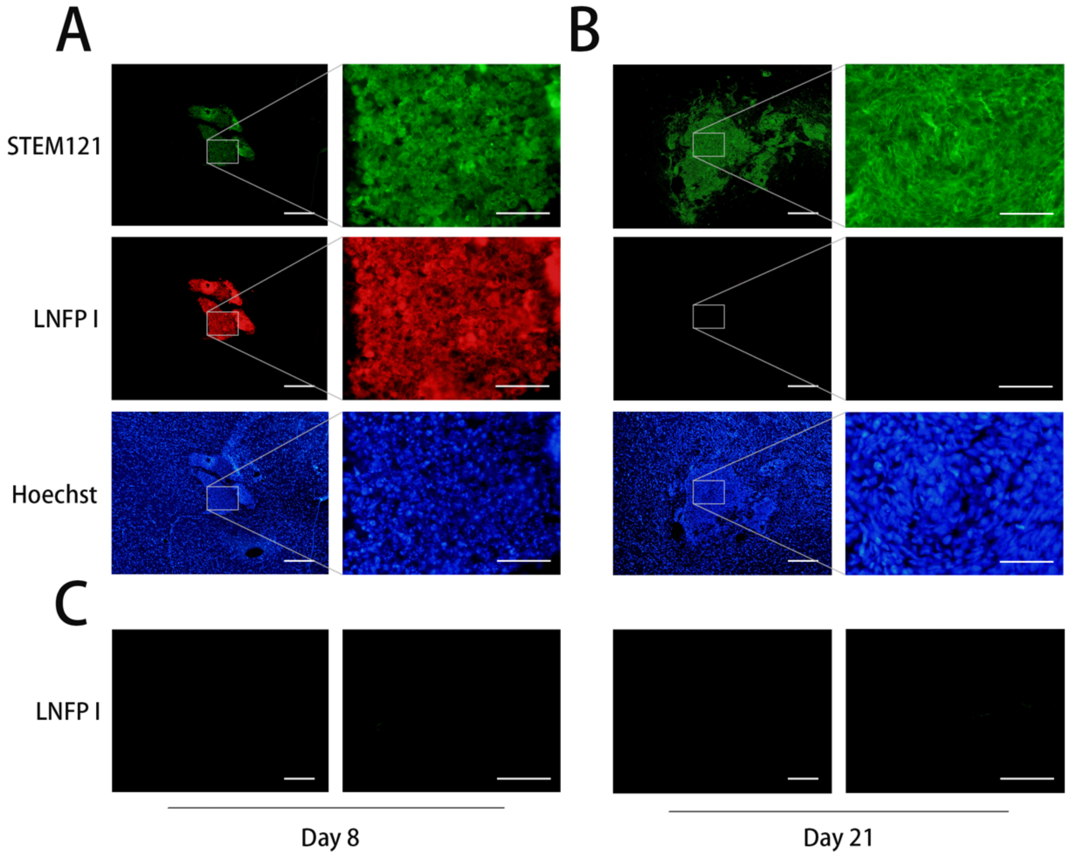

Supplement: Supplementary file 1 [file JNC-153-759-s001.pdf]
